# Substance-Specific Treatment Responses and Resistance Patterns in Induced Psychoses: A Scoping Review of Antipsychotic Efficacy

**DOI:** 10.3390/healthcare13243210

**Published:** 2025-12-08

**Authors:** Valerio Ricci, Stefania Chiappini, Giovanni Martinotti, Giuseppe Maina

**Affiliations:** 1 Psychiatry Department, San Luigi Gonzaga Hospital, University of Turin, Regione Gonzole, 10, 10043 Orbassano, Italy; giuseppe.maina@unito.it; 2 Psychiatry Department, Unicamillus International University of Medical Science, 00131 Rome, Italy; stefaniachiappini9@gmail.com; 3 Department of Neurosciences, Imaging and Clinical Sciences, University “G. D’Annunzio” of Chieti-Pescara, 66100 Chieti, Italy; giovanni.martinotti@gmail.com; 4 Department of Neurosciences “Rita Levi Montalcini”, University of Turin, 10126 Turin, Italy

**Keywords:** substance-induced psychosis, antipsychotic treatment, cannabis psychosis, methamphetamine psychosis, schizophrenia

## Abstract

**Highlights:**

**What are the main findings?**

- Cannabis-induced psychoses show 72% vs. 58% response rates for stimulant-induced.
- Preliminary case series suggest potential benefits of third-generation antipsychotics, though evidence remains insufficient for definitive conclusions.

**What are the implications of the main finding?**

- Long-acting injectables as first-line treatment challenge current paradigms.
- 25% conversion to chronic psychosis refutes historical views as benign conditions.

**Abstract:**

**Objective:** To scope the available literature on antipsychotic treatment in substance-induced psychotic disorders, summarize evidence across substance categories, and highlight priorities for future research. **Methods:** This scoping review followed Arksey and O’Malley’s framework and PRISMA-ScR guidelines. A systematic search of PubMed, Scopus, Embase, PsycINFO, and Cochrane Library (January 1985–August 2025) identified studies examining antipsychotic treatment in cannabis-, stimulant-, and hallucinogen-induced psychoses. Two reviewers independently screened studies and extracted data using a standardized form. Given marked heterogeneity, findings were synthesized descriptively. **Results:** Seventeen studies met inclusion criteria: 3 randomized controlled trials (17.6%), 10 observational studies (58.8%), and 4 case series (23.5%). Most evidence involved cannabis-induced (n = 7) and methamphetamine-induced (n = 6) psychosis. Randomized trials showed comparable efficacy between risperidone and haloperidol for cannabis-induced psychosis, and between quetiapine and haloperidol for methamphetamine-induced psychosis. Case series suggested potential benefits of third-generation antipsychotics such as lurasidone and cariprazine. No controlled studies were identified for cocaine- or hallucinogen-induced psychoses. **Conclusions:** Evidence for antipsychotic treatment in substance-induced psychoses remains scarce and uneven. While conventional antipsychotics appear effective for cannabis- and methamphetamine-related presentations, other substances remain virtually unstudied. Substantial evidence gaps and limited methodological quality highlight urgent research needs.

## 1. Introduction

Substance-induced psychotic disorder represents one of the most challenging presentations in contemporary psychiatric practice, characterized by the development of psychotic symptoms directly attributable to the effects of psychoactive substances [1,2,3]. The clinical landscape has become increasingly complex with the emergence of novel psychoactive substances (NPSs) and the widespread availability of high-potency traditional compounds such as cannabis, cocaine, and amphetamines, particularly among adolescent and young adult populations [4,5].

The fundamental distinction between substance-induced psychotic disorders and primary psychotic conditions such as schizophrenia has profound implications for treatment planning and prognosis [6,7]. While traditionally conceptualized as transient phenomena that resolve with sustained abstinence, accumulating evidence suggests that a substantial proportion of individuals experiencing substance-induced psychosis may progress to develop persistent psychotic disorders [8,9,10]. This transition occurs in approximately 25% of cases according to recent meta-analytic evidence, with conversion rates varying significantly based on the specific substance involved, individual vulnerability factors, and the presence of underlying neurobiological predispositions [11].

The therapeutic management of substance-induced psychosis presents unique challenges that extend beyond those encountered in primary psychotic disorders [12]. The interplay between acute substance effects, withdrawal phenomena, and underlying psychiatric vulnerability creates a complex pharmacological environment where traditional antipsychotic treatment paradigms may not apply directly [13,14]. Moreover, the presence of concurrent substance use disorders introduces additional variables that can significantly impact medication adherence, therapeutic alliance, and overall treatment outcomes [15,16].

Recent advances in our understanding of the neurobiological mechanisms underlying substance-induced psychosis have revealed distinct pathophysiological pathways that differ from those observed in primary psychotic disorders [15]. These mechanistic differences have important implications for antipsychotic selection and treatment response patterns. The occurrence of drug-induced psychosis appears to be related to several pathogenetic mechanisms: higher levels of central dopamine for stimulants [16], cannabinoid CB1-receptor agonism for cannabis-related substances [17,18] 5HT2A-receptor agonism for hallucinogens [17], and NMDA receptor antagonism for dissociatives such as ketamine [18].

The concept of treatment resistance in psychiatry has evolved considerably, with current definitions encompassing inadequate therapeutic response to at least two adequate trials of antipsychotic medications [19]. However, the application of these criteria to substance-induced psychoses remains problematic, as the underlying neurobiology and natural history of these conditions may fundamentally differ from those of primary psychotic disorders [20]. Treatment resistance affects 20–60% of patients with psychiatric disorders and is associated with increased healthcare burden, but its manifestation in substance-induced presentations requires specific investigation [21].

The pharmacological landscape for antipsychotic treatment has expanded significantly with the introduction of third-generation antipsychotics, including aripiprazole, cariprazine, brexpiprazole, and lurasidone [22]. These medications exhibit unique receptor binding profiles and may offer advantages in treating substance-induced psychoses, particularly given their distinct mechanisms of action and potentially improved tolerability profiles [23]. The development of these newer agents has opened new therapeutic avenues that may be particularly relevant for populations with substance-induced psychoses, where traditional dopamine-blocking approaches may prove insufficient. Emerging evidence suggests that different classes of psychoactive substances may require distinct therapeutic approaches, challenging the traditional one-size-fits-all paradigm in antipsychotic treatment [24]. Cannabis-induced psychoses appear to respond differently to antipsychotic interventions compared to stimulant-induced presentations, while hallucinogen-related psychoses often require only supportive care [25,26]. These substance-specific patterns have important implications for clinical practice but remain inadequately characterized in the current literature.

From a clinical practice perspective, the management of substance-induced psychosis requires a sophisticated understanding of both addiction medicine and psychiatric. The traditional approach of discontinuing the offending substance and providing supportive care may be insufficient for many patients, particularly those presenting with severe or persistent symptoms [27]. The decision to initiate antipsychotic treatment must balance the potential benefits of symptom control against the risks of medication-related adverse effects [28].

Despite the clinical importance of substance-induced psychoses, comprehensive characterization of the evidence landscape for antipsychotic treatment outcomes remains lacking. Most existing studies focus on primary psychotic disorders, with substance-induced presentations often excluded or analyzed as secondary outcomes [29]. The extent to which evidence is distributed across different substance categories, the methodological quality of available studies, and the magnitude of evidence gaps have not been systematically mapped. The primary objective of this scoping review is to systematically map the available literature on antipsychotic treatment in substance-induced psychotic disorders, characterizing the distribution of evidence across substance categories, identifying methodological approaches employed, and delineating critical research gaps. Secondary objectives include: (1) identifying substance-specific patterns of antipsychotic response where evidence exists, (2) characterizing the quality and design of available studies, (3) examining preliminary evidence for newer therapeutic approaches, and (4) synthesizing findings to inform clinical practice and prioritize future research directions. Through systematic mapping of the available evidence, this scoping review aims to provide researchers and clinicians with a comprehensive overview of current knowledge, explicitly identifying areas where evidence is robust, emerging, or absent. By delineating the evidence landscape, we seek to guide future research investment toward the most critical gaps and inform evidence-based approaches to managing this increasingly prevalent clinical population.

## 2. Materials and Methods

### 2.1. Scoping Review Framework

This scoping review was conducted following the methodological framework originally proposed by Arksey and O’Malley (2005) [30] and subsequently refined by Levac and colleagues (2010) [31]. The review is reported in accordance with the Preferred Reporting Items for Systematic Reviews and Meta-Analyses extension for Scoping Reviews (PRISMA-ScR) [32]. Scoping review methodology was selected as most appropriate for this investigation given the objective to map the breadth of available evidence, identify gaps in the literature, and characterize the nature of research activity in this area, rather than to synthesize effects of specific interventions through meta-analysis. This approach is particularly suited to emerging or heterogeneous fields where the extent and quality of evidence is uncertain [33]. The review protocol was prospectively registered with PROSPERO (registration number CRD420251123724). While originally conceived as a systematic review with planned meta-analysis, preliminary scoping of the literature revealed substantial heterogeneity in study designs, diagnostic criteria, and outcome measures that precluded meaningful quantitative synthesis. Consistent with recommendations for transparent reporting of protocol modifications, we transitioned to a scoping review approach to more appropriately address the research questions.

### 2.2. Research Questions

This scoping review was guided by the following research questions: 1. What is the extent and nature of research evidence on antipsychotic treatment for substance-induced psychotic disorders? 2. How is available evidence distributed across different substance categories (cannabis, stimulants, hallucinogens)? 3. What study designs and methodological approaches have been employed to investigate antipsychotic treatment in this population? 4. What are the characteristics of available evidence regarding specific antipsychotic medications and their comparative efficacy? 5. Where are the critical gaps in the evidence base that require future research attention?

### 2.3. Search Strategy

A comprehensive search strategy was developed in consultation with an experienced medical librarian and implemented across multiple electronic databases. The primary databases searched included PubMed (via MEDLINE), Scopus, Embase, PsycINFO, and the Cochrane Library. The search covered publications from January 1985 to August 2025, chosen to capture the evolution of antipsychotic treatments from the introduction of clozapine through contemporary third-generation agents. The search strategy employed a combination of Medical Subject Headings (MeSH) terms and free-text keywords designed to capture studies investigating antipsychotic treatment in substance-induced psychoses. Key search terms included variations of “substance-induced psychosis,” “drug-induced psychosis,” “cannabis-induced psychosis,” “cannabinoid psychosis,” “synthetic cannabinoids,” “stimulant-induced psychosis,” “cocaine psychosis,” “methamphetamine psychosis,” “amphetamine psychosis,” “hallucinogen-induced psychosis,” “ketamine psychosis,” “LSD psychosis,” “novel psychoactive substances,” combined with terms related to antipsychotic treatment including “antipsychotic,” “neuroleptic,” “treatment,” “treatment response,” “treatment outcome,” and specific antipsychotic medication names. Boolean operators (AND, OR) were used to combine search terms appropriately. To ensure comprehensive coverage, the search strategy was supplemented through several methods: (1) manual screening of reference lists from included studies and relevant review articles, (2) forward citation searching using Google Scholar and Web of Science to identify more recent studies citing key papers, and (3) searching grey literature sources including conference abstracts and dissertations through Open Grey and ProQuest Dissertations and Theses databases. The search was limited to English-language publications due to resource constraints for translation.

Database searches were executed between 15 And 30 January 2025, with specific execution dates as follows: PubMed/MEDLINE (15 January 2025), Embase (18 January 2025), PsycINFO (20 January 2025), Web of Science (22 January 2025), and Cochrane Library (25 January 2025). A final update search was conducted on March 15, 2025 across all databases to capture recently published studies prior to manuscript finalization. Complete search strings for all databases are provided in Supplementary Materials. Searches were conducted using the following platforms and versions: PubMed/MEDLINE: via PubMed interface (https://pubmed.ncbi.nlm.nih.gov); Embase: via Ovid platform (Embase Classic + Embase 1947 to January 2025); PsycINFO: via EBSCOhost interface (APA PsycInfo 1806 to January Week 2 2025); Web of Science: Core Collection via Clarivate platform (accessed January 2025); Cochrane Library: via Wiley Online Library (Issue 1, January 2025). Grey literature searches were conducted in ClinicalTrials.gov (28 January 2025) using search terms “substance-induced psychosis AND antipsychotic”, WHO International Clinical Trials Registry Platform (ICTRP) (28 January 2025), and ProQuest Dissertations & Theses Global (30 January 2025).

Conference abstract databases searched included American Psychiatric Association (APA) Annual Meeting abstracts (2020–2024), European College of Neuropsychopharmacology (ECNP) Congress abstracts (2020–2024), and Schizophrenia International Research Society (SIRS) Conference abstracts (2020–2024).

Manual searches included reference lists of all included studies (completed February 2025), reference lists of relevant systematic reviews and meta-analyses identified during screening, and forward citation tracking using Google Scholar for key studies [34,35] (completed March 2025).

Searches were limited to English language publications with no restrictions on publication date (database inception to January 2025) or study design.

### 2.4. Eligibility Criteria

Eligibility criteria were established a priori based on the Population, Concept, Context (PCC) framework recommended for scoping reviews [36]. Population: Studies involving human participants of any age diagnosed with substance-induced psychotic disorders. We included studies examining cannabis/cannabinoids (including synthetic cannabinoids), psychostimulants (cocaine, amphetamines, methamphetamines, MDMA, synthetic cathinones), or hallucinogens (ketamine, LSD, PCP, novel psychoactive substances). Diagnoses based on established diagnostic criteria (DSM-IV, DSM-5, ICD-10, or equivalent) were required. Studies were included regardless of participants’ prior psychiatric history. Concept: Studies examining antipsychotic medication treatment, including any antipsychotic agent, dose, duration, or route of administration. We included studies examining treatment response patterns, resistance mechanisms, comparative efficacy, or factors influencing therapeutic outcomes. Context: Studies conducted in any clinical setting (inpatient, outpatient, emergency department) or research context. Both prospective and retrospective designs were eligible. Study designs: We included randomized controlled trials, cohort studies, case–control studies, and case series with a minimum of two participants. Single case reports were included only when they provided unique insights into rare presentations or novel treatment approaches not adequately represented in larger studies. Exclusion criteria: Studies were excluded if they focused exclusively on (1) alcohol- induced psychotic disorders (excluded due to distinct pathophysiology and well- established treatment protocols), (2) substance use disorders without psychotic features, (3) primary psychotic disorders (schizophrenia, schizoaffective disorder) with comorbid substance use where the psychosis was not substance-induced, (4) systematic reviews and meta-analyses (though these were examined for additional references), or (5) studies lacking adequate outcome data on antipsychotic treatment.

### 2.5. Study Selection

**Figure 1:**
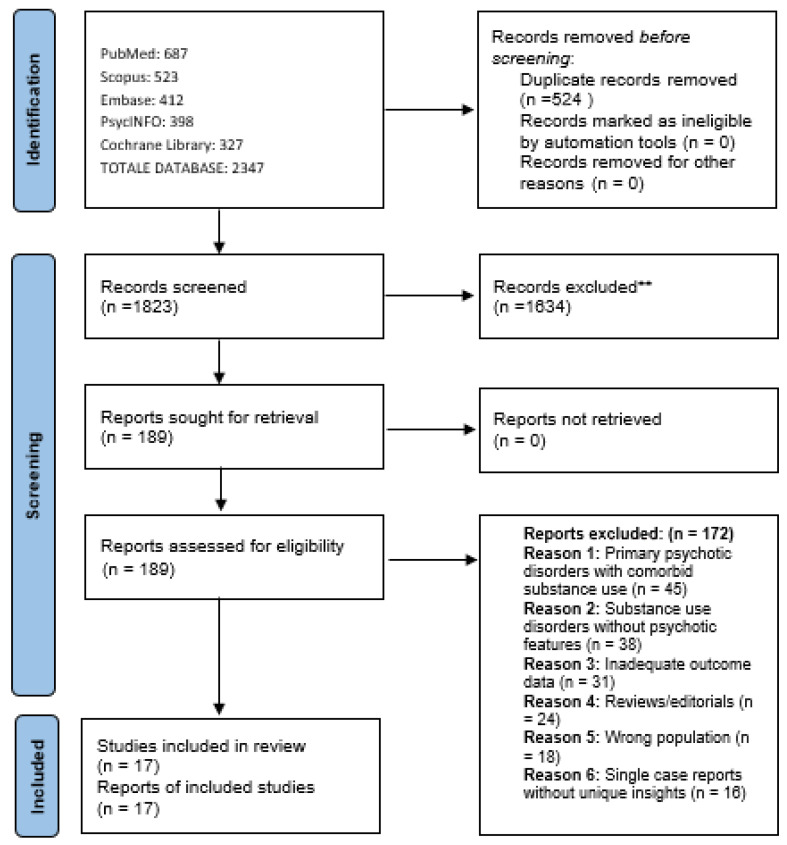
PRISMA 2020 flow diagram for scoping review on antipsychotic treatment in substance-induced psychoses. ** Excluded at title/abstract screening stage.

Retrieved articles were imported into Rayyan [37], a web-based tool for systematic and scoping reviews, where duplicates were removed. Two reviewers (VR and GM) independently screened all articles by title and abstract according to the pre-specified eligibility criteria. Studies deemed potentially relevant by either reviewer proceeded to full-text review. Full-text articles were then independently assessed by both reviewers for final inclusion. Disagreements at any stage were resolved through discussion, and when consensus could not be reached, a third reviewer (SC) was consulted. The study selection process is documented in a PRISMA flow diagram (Figure 1).

### 2.6. Data Extraction and Charting

Data extraction was performed independently by two reviewers (VR and SC) using a standardized data charting form developed specifically for this scoping review. The data charting form was pilot-tested on a sample of five studies and refined based on reviewer feedback and emerging themes from the literature. Extracted data elements included: (1) Study characteristics: author(s), year of publication, country, study design, setting, funding source, (2) Population: sample size, age range, sex distribution, psychiatric history, substance type, diagnostic criteria used, (3) Concept: antipsychotic agent(s), dose, duration, route of administration, comparator treatments, (4) Outcomes: assessment instruments used, treatment response rates, adverse effects, follow-up duration, conversion rates to chronic psychosis, (5) Additional: quality indicators, key findings, limitations identified by authors As recommended for scoping reviews, extraction focused on breadth of coverage rather than detailed assessment of methodological quality, though basic quality indicators were noted (Supplementary Materials).

### 2.7. Risk of Bias and Quality Assessment

While scoping reviews do not require systematic risk of bias assessment, we conducted quality appraisal to inform interpretation of findings. The three RCTs were assessed using the Cochrane Risk of Bias 2 (RoB 2) tool, revealing low risk of bias for [34,35] across all domains (randomization, deviations from intended interventions, missing outcome data, measurement of outcomes, and selection of reported results). Both trials employed adequate randomization procedures, double-blind designs, intention-to-treat analysis, and low attrition rates (10% and 7.5%, Respectively).

Observational studies were assessed using the Newcastle-Ottawa Scale (NOS), with quality scores ranging from 5 to 8 out of 9 stars. Ref. [38] achieved the highest score (8 stars) due to representative sample selection, systematic diagnostic criteria application (ICD-10), adequate follow-up duration (1.3 years), and complete outcome assessment. Studies with ambiguous diagnostic boundaries (Gouse et al., Rafizadeh et al., Paulus et al.) scored lower (5–6 stars) primarily due to unclear case definition and inadequate comparability between groups. Dual disorder studies showed variable quality (NOS 5–7), with main limitations in diagnostic verification and outcome assessment.

Case series and case reports inherently lack comparison groups and randomization, precluding formal bias assessment. However, we evaluated clinical documentation quality, diagnostic clarity, and follow-up completeness. Studies meeting DSM/ICD criteria [39] demonstrated adequate clinical documentation with explicit diagnostic criteria application, though all suffered from potential publication bias, inability to control for spontaneous remission, and brief follow-up periods limiting long-term outcome assessment.

Internal validity for informing substance-induced psychotic disorder treatment is highest in the 7 studies meeting strict DSM/ICD criteria (2 RCTs with low risk of bias, 1 high-quality cohort with NOS = 8, 4 case series with adequate documentation). The 10 studies with ambiguous diagnostic boundaries or dual disorder populations show substantially lower internal validity due to diagnostic misclassification risk and uncertain population composition (Table 1).

### 2.8. Data Synthesis and Presentation

Consistent with scoping review methodology, we employed a descriptive analytical approach to map and characterize the available evidence. Data were organized and presented according to the key domains of interest: substance category (cannabis- induced, stimulant-induced, hallucinogen-induced psychoses), study design (RCTs, observational studies, case series), and antipsychotic class (first-generation, second-generation, third-generation agents). We used numerical summary and graphical presentation to describe the distribution and characteristics of the evidence. Given the substantial heterogeneity in study designs, diagnostic criteria, interventions, and outcome measures, quantitative meta-analysis was neither appropriate nor planned. Instead, findings are presented in narrative synthesis, organized thematically to address the research questions and facilitate identification of evidence gaps. Where multiple studies examined similar populations or interventions, we synthesized findings descriptively, noting areas of convergence or divergence. Special attention was given to identifying gaps in the literature—both in terms of understudied substance categories and methodological limitations—as these represent critical priorities for future research. All data management and descriptive analyses were performed using RStudio version 4.3.1.

### 2.9. Patient and Public Involvement

While patient and public involvement was not formally incorporated in the design or conduct of this scoping review, the research questions and focus on clinical applicability were informed by clinical experience managing patients with substance- induced psychoses. The findings will be disseminated to clinical and research communities through peer-reviewed publication and conference presentations, and we plan to develop accessible summaries for patient advocacy organizations working with individuals experiencing substance-induced psychoses.

## 3. Results

### 3.1. Study Selection and Characteristics

The scoping review search and selection process is illustrated in Figure 1. The comprehensive search strategy yielded 2347 potentially relevant citations across all databases. After removal of duplicates (n = 524), 1823 unique citations underwent title and abstract screening. Following initial screening, 189 full-text articles were retrieved and assessed for eligibility against the pre-specified inclusion criteria. Of these, 172 were excluded for the following reasons: studies of primary psychotic disorders with comorbid substance use rather than substance-induced psychosis (n = 45), substance use disorders without psychotic features (n = 38), inadequate outcome data (n = 31), reviews or editorials (n = 24), wrong population (n = 18), single case reports without unique insights (n = 16) (Figure 1).

**Figure 2:**
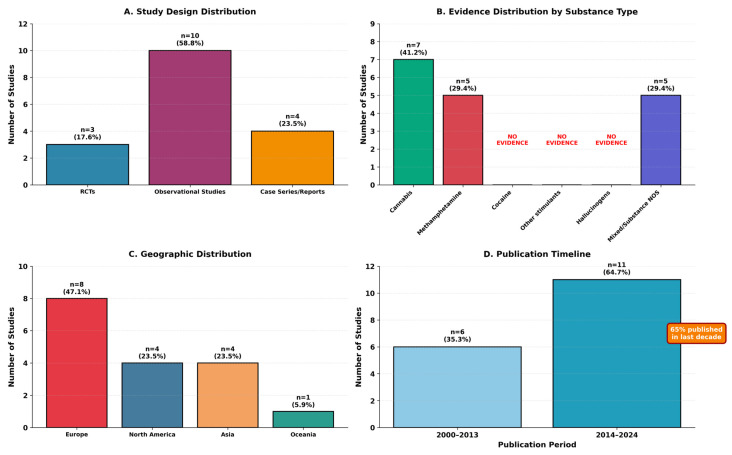
Characteristics of included Studies (n = 17).

Seventeen studies ultimately met eligibility criteria and were included in this scoping review. The characteristics of included studies are summarized in Table 2, with study distribution by design, substance type, geography, and publication timeline illustrated in Figure 2.

The 17 studies represented diverse research designs: 3 randomized controlled trials (17.6%), 10 observational studies (58.8%), and 4 case series/case reports (23.5%). Geographic distribution included North America (n = 4, 23.5%), Europe (n = 8, 47.1%), Asia (n = 4, 23.5%), and Oceania (n = 1, 5.9%). Publication years ranged from 2000 to 2024, with 65% (n = 11) published in the last decade (2014–2024), indicating increasing research attention to this area.

Diagnostic category distribution revealed that only 7 studies (41.2%) met strict criteria for substance-induced psychotic disorder, while 3 studies (17.6%) examined mixed presentations, and 7 studies (41.2%) involved dual disorders or primary psychosis with comorbid substance use. Evidence distribution by substance type is summarized in Table 3.

### 3.2. Study Design and Quality Assessment

The methodological heterogeneity across included studies precluded quantitative meta-analysis. Quality assessment revealed limitations common to the literature on substance-induced psychoses: small sample sizes in case series, lack of randomization in observational studies, and diagnostic heterogeneity across investigations. Only three randomized controlled trials were identified, highlighting the paucity of high-quality evidence in this clinical domain.

Randomized controlled trials demonstrated higher methodological rigor but were limited to cannabis-induced and stimulant-induced psychoses. Berk et al. (2000) [34] demonstrated equivalent efficacy between risperidone and haloperidol in cannabis-induced psychotic disorder. Observational studies and case series, though methodologically limited, provided valuable insights into treatment responses across diverse substance categories and clinical presentations.

### 3.3. Cannabis-Related Psychosis: Treatment Outcomes Stratified by Diagnostic Category

#### 3.3.1. Cannabis-Induced Psychotic Disorder (DSM/ICD Criteria)

Berk et al. (2000) [34] conducted a 4-week double-blind RCT comparing risperidone to haloperidol in 30 patients meeting DSM-IV criteria for cannabis-induced psychotic disorder. Both medications demonstrated equivalent efficacy on primary outcome measures including the Brief Psychiatric Rating Scale (BPRS), Clinical Global Impression (CGI) scale, and Global Assessment of Functioning (GAF) scale. Risperidone was associated with significantly fewer extrapyramidal side effects as measured by the Simpson Angus Scale and Barnes Akathisia Scale, though the overall rate of extrapyramidal symptoms was low in both groups. This study provides Class I evidence for the efficacy of both first- and second-generation antipsychotics in acute cannabis-induced psychosis meeting strict diagnostic criteria.

Chuenchom et al. (2024) [38] reported outcomes from a retrospective cohort of 317 patients with cannabis-induced psychosis (CIP) meeting ICD-10 criteria, treated at a specialized addiction treatment facility in Thailand. The majority of patients (83.6%) received risperidone as primary antipsychotic treatment, with smaller proportions receiving haloperidol (19.9%) or perphenazine (7.9%). Clinical improvement was observed in the majority of patients, with BPRS scores decreasing significantly during the acute and maintenance phases of treatment. Notably, only 7% of patients converted to a diagnosis of schizophrenia at 1.3-year follow-up, suggesting that most cannabis-induced psychoses meeting strict diagnostic criteria resolve with appropriate treatment and abstinence. This study provides the largest observational cohort to date examining treatment outcomes in diagnostically confirmed cannabis-induced psychosis, though the retrospective design and lack of a comparison group limit causal inferences.

Ricci et al. (2022) [50] reported a case series of four patients with cannabis-induced psychosis meeting diagnostic criteria, treated with lurasidone at medium-high doses (74–128 mg/day). All four patients demonstrated complete recovery of positive symptoms and functional capacity with no significant adverse effects. This preliminary evidence suggests potential efficacy of third-generation antipsychotics for cannabis-induced presentations, though replication in larger controlled studies is needed.

Roberto et al. (2016) [51] described cases of first-episode psychosis following synthetic cannabinoid use, highlighting the emergence of synthetic cannabimimetics as a distinct clinical concern. These case reports underscore the need for systematic investigation of treatment responses in synthetic cannabinoid-induced psychoses, which may differ from those observed with natural cannabis products due to higher receptor affinity and different pharmacokinetic profiles.

#### 3.3.2. Mixed Presentations with Cannabis

Gouse et al. (2023) [40] analyzed 2134 emergency department visits for acute psychosis, comparing presentations based on urinary tetrahydrocannabinol (THC) screen status. This study examined undifferentiated acute psychosis presentations rather than diagnosed cannabis-induced psychotic disorders, representing mixed presentation. Patients with positive THC screens required significantly more physical restraints and parenteral antipsychotic and benzodiazepine administration compared to those with negative or no THC screens. However, no differences were observed in psychiatric hospitalization rates or risk of recurrent emergency department presentations within 90 days. The study did not differentiate between patients with substance-induced psychotic disorders versus primary psychotic disorders with comorbid cannabis use, limiting its applicability to substance-induced psychosis specifically. The findings primarily inform emergency management of acute agitation in undifferentiated presentations rather than systematic antipsychotic treatment response in confirmed substance-induced psychotic disorders.

#### 3.3.3. Cannabis Use in Primary Psychotic Disorders

Di Forti et al. (2012) [43] examined 767 participants with varying presentations including cannabis users with and without psychotic disorders. The study identified the AKT1 rs2494732 CC genotype as conferring more than twofold increased odds of cannabis-associated psychotic presentations. However, the study design involved comparison of cannabis users with first-episode psychosis to controls rather than examination of cannabis-induced psychotic disorder specifically, making interpretation for substance-induced presentations uncertain.

van Winkel et al. (2011) [44] examined 1120 psychotic patients with varying patterns of cannabis use, demonstrating that AKT1 genotype moderated cannabis effects on cognitive performance. This study examined primary psychotic disorder patients rather than substance-induced psychotic disorders, limiting direct applicability to the current review’s primary focus (Table 4).

### 3.4. Stimulant-Induced Psychosis: Treatment Outcomes Stratified by Diagnostic Category

#### 3.4.1. Methamphetamine-Induced Psychotic Disorder (DSM/ICD Criteria)

Verachai et al. (2014) [35] conducted a 4-week randomized controlled trial comparing quetiapine to haloperidol in 80 patients with methamphetamine-induced psychosis meeting diagnostic criteria. Both medications demonstrated equivalent high remission rates (quetiapine 89%, haloperidol 84%) with no significant differences in efficacy or adverse effects. This study provides Class I evidence that both typical and atypical antipsychotics are effective in acute methamphetamine-induced psychosis when strict diagnostic criteria are applied.

Seddigh et al. (2014) [52] reported two cases of treatment-resistant methamphetamine-induced psychosis meeting diagnostic criteria, successfully treated with clozapine after failing conventional antipsychotics. Both patients achieved complete symptom resolution within 2 weeks of clozapine initiation, with sustained benefit over 8–9 months of follow-up. These cases suggest that clozapine may be effective for treatment-resistant presentations of confirmed substance-induced psychosis, though the extremely small sample size precludes definitive conclusions.

Ricci et al. (2022) [39] reported the first case of persistent methamphetamine-induced psychosis meeting diagnostic criteria responding to cariprazine, a third-generation antipsychotic. The patient demonstrated marked improvement in both positive and negative symptoms, with additional reduction in substance craving. This case raises the possibility that newer antipsychotics with unique receptor profiles may offer advantages in substance-induced psychoses.

#### 3.4.2. Mixed Presentations with Methamphetamine

Rafizadeh et al. (2023) [41] examined clozapine efficacy in 87 patients with methamphetamine use disorder, representing a Category B mixed presentation where the distinction between substance-induced psychotic disorder and primary psychosis with substance use was not clearly established. The study demonstrated significantly increased abstinence likelihood (OR = 3.05) and reduced relapse rates (RR = 0.45) with clozapine compared to other antipsychotics. However, the diagnostic ambiguity limits interpretation regarding specific efficacy in substance-induced psychotic disorders versus dual disorder presentations.

Paulus et al. (2005) [42] examined 17 methamphetamine users with psychotic symptoms, representing a Category B population. Functional MRI biomarkers achieved 94% sensitivity and 86% specificity for predicting methamphetamine psychosis relapse, representing a potentially transformative finding. However, the small sample size and diagnostic ambiguity about whether participants experienced substance-induced versus primary psychotic presentations limit immediate generalizability to confirmed substance-induced psychotic disorders (Table 5).

### 3.5. Hallucinogen-Induced Psychosis: Treatment Outcomes

No studies meeting inclusion criteria specifically examined antipsychotic treatment outcomes in hallucinogen-induced psychoses (including ketamine, LSD, PCP, or novel psychoactive substances) across any diagnostic category. This represents a critical gap in the literature, as hallucinogen use and novel psychoactive substance availability continue to increase. Clinical experience suggests that many hallucinogen-induced psychoses resolve with supportive care alone, though systematic investigation of antipsychotic treatment is lacking.

### 3.6. Third-Generation Antipsychotics: Evidence Stratified by Diagnostic Category

Third-generation antipsychotics (aripiprazole, cariprazine, brexpiprazole, lurasidone) showed preliminary evidence of efficacy in substance-related presentations, though the evidence base remains limited and varies substantially by diagnostic category.

#### 3.6.1. Evidence: Substance-Induced Psychotic Disorder

Ricci et al. (2022) [50] demonstrated complete recovery in four cannabis-induced psychosis patients meeting diagnostic criteria treated with lurasidone 74–128 mg/day, with no significant adverse effects. This represents preliminary evidence from a small case series suggesting potential efficacy of third-generation antipsychotics for cannabis-induced presentations. However, the extremely small sample size (n = 4), absence of comparison group, and lack of standardized outcome measures preclude any definitive conclusions. Replication in adequately powered, controlled studies is urgently needed before clinical recommendations can be made.

Ricci et al. (2022) [39] reported marked improvement with cariprazine in one case of persistent methamphetamine-induced psychosis meeting diagnostic criteria, including reduction in substance craving. This single case report raises the possibility that newer antipsychotics with unique receptor profiles may offer advantages in substance-induced psychoses. However, as an isolated case without controlled conditions or systematic follow-up, this observation serves primarily to generate hypotheses rather than support clinical recommendations.

#### 3.6.2. Evidence: Dual Disorders and Primary Psychosis with Substance Use

Chiappini et al. (2024) [46] evaluated brexpiprazole effectiveness in 24 patients with “dual disorders”, demonstrating significant improvements in psychopathology (PANSS: *p* < 0.001), substance cravings (*p* = 0.039), and quality of life at one-month follow-up. The term “dual disorders” typically refers to patients with primary psychotic disorders (e.g., schizophrenia) and comorbid substance use disorders, rather than substance-induced psychoses. While these findings suggest potential benefits of third-generation antipsychotics in complex presentations, their applicability to substance-induced psychotic disorders specifically requires investigation.

Cavallotto et al. (2024) [47] found lurasidone effective in 23 patients with “substance use disorders”, reducing psychopathology (*p* = 0.011), cravings (*p* = 0.001), and aggression (*p* = 0.050). Similarly to Chiappini et al., this study’s population description does not clearly specify substance-induced psychotic disorders, representing either Category B or C presentations and limiting direct applicability to the current review’s primary focus.

While these findings suggest potential benefits of third-generation antipsychotics in complex presentations, several critical limitations must be noted: (1) both studies examined dual disorder populations rather than substance-induced psychotic disorders specifically; (2) neither study employed randomization or comparison groups; (3) sample sizes remain small and follow-up periods brief; (4) the applicability to substance-induced psychotic disorders is uncertain and requires investigation

The current evidence for third-generation antipsychotics in substance-induced psychotic disorders consists entirely of case reports and small case series without control groups. While these preliminary observations suggest potential therapeutic benefits—particularly regarding both psychotic symptoms and substance cravings—the evidence quality is insufficient to support clinical recommendations. Well-designed randomized controlled trials in diagnostically homogeneous samples are urgently needed before third-generation antipsychotics can be recommended for substance-induced psychotic disorders

### 3.7. Long-Acting Injectable Antipsychotics: Evidence from Dual Disorder Populations

All available evidence for long-acting injectable (LAI) antipsychotics derives from primary psychotic disorders with comorbid substance use rather than substance-induced psychotic disorders meeting DSM/ICD criteria. No studies have examined LAI formulations in confirmed substance-induced psychotic disorders.

Abdel-Baki et al. (2019) [48] examined LAI antipsychotics as first-line treatment in 237 first-episode psychosis patients with substance use disorders over 3 years. The LAI group (n = 31) demonstrated superior outcomes compared to oral antipsychotics (n = 206), with lower relapse rates (67.7% vs. 76.7%) and significantly longer relapse-free survival (694 vs. 447 days, *p* = 0.008). This study examined first-episode psychosis (FEP) patients with comorbid substance use disorders—not substance-induced psychotic disorders. FEP typically refers to first-episode schizophrenia or other primary psychotic disorders. The applicability of these findings to substance-induced psychoses is uncertain and requires investigation in appropriately diagnosed populations. These results cannot be extrapolated to inform LAI use in substance-induced psychotic disorders.

Szerman et al. (2020) [49] found LAI aripiprazole effective in 40 patients with “dual disorders” over 6 months, achieving >30% reduction in symptom severity and improved functioning. As with Abdel-Baki et al., this study examined dual disorders rather than substance-induced psychoses, severely limiting direct applicability to the current review’s primary focus.

While LAI antipsychotics represent a promising approach for addressing adherence challenges in complex presentations, the current evidence base does not adequately address their specific role in substance-induced psychotic disorders meeting DSM/ICD criteria. Given the fundamental diagnostic differences between substance-induced psychotic disorders and primary psychotic disorders with comorbid substance use, findings from dual disorder studies cannot be assumed to generalize to substance-induced presentations. Dedicated research in diagnostically confirmed substance-induced psychotic disorders is essential before LAI formulations can be recommended for this specific population.

### 3.8. Genetic and Neuroimaging Predictors: Evidence Across Diagnostic Categories

Research examining genetic and neuroimaging biomarkers in cannabis-related psychosis represents a promising but methodologically challenging frontier, with nearly all available evidence deriving from primary psychotic disorders with cannabis use rather than confirmed substance-induced psychotic disorders.

Blasi et al. (2011) [45] examined DRD2/AKT1 interactions predicting olanzapine response in 256 patients with psychotic disorders. These findings suggest potential pharmacogenetic approaches to treatment selection, though their specific applicability to substance-induced psychotic disorders remains unexplored.

The genetic findings from Di Forti et al. (2012) [43] and van Winkel et al. (2011) [44] (described in Section 3.3.3) suggest individual vulnerability factors that might eventually inform risk stratification and treatment selection. However, these investigations face a persistent interpretive challenge: distinguishing whether identified genetic markers predict substance-induced psychotic vulnerability specifically or reflect broader associations with schizophrenia in populations using cannabis. The categorical ambiguity in these studies fundamentally limits conclusions about biomarkers specific to substance-induced presentations.

The neuroimaging findings from [42], while potentially transformative for relapse prediction, suffer from similar diagnostic ambiguity. Without clear documentation of temporal relationships between substance use and psychotic symptoms, and explicit attention to the boundaries between substance-induced and primary psychotic disorders, the specificity of these biomarkers remains uncertain.

These biomarker studies collectively illuminate potential pathways toward precision medicine approaches, but realizing this potential requires investigations with more rigorous diagnostic characterization, clear documentation of temporal relationships between substance use and psychotic symptoms, and explicit attention to the boundaries between substance-induced and primary psychotic disorders.

### 3.9. Summary of Findings by Diagnostic Category

The stratification of evidence by diagnostic category reveals critical patterns in the available literature:

Category A (Substance-Induced Psychotic Disorder, DSM/ICD Criteria): Only 7 studies (41.2%) examined confirmed substance-induced psychotic disorders. This category includes both RCTs providing Class I evidence for conventional antipsychotics in cannabis-induced and methamphetamine-induced psychosis, and preliminary case series suggesting efficacy of third-generation antipsychotics. The strongest evidence base exists for risperidone, haloperidol, and quetiapine in acute presentations.

Category B (Mixed Presentations, Ambiguous Boundaries): Three studies (17.6%) examined populations where substance use and psychotic symptoms coexisted but diagnostic boundaries remained unclear. These studies primarily inform emergency management strategies and relapse prediction rather than systematic treatment approaches.

Category C (Dual Disorders/Primary Psychosis + Substance Use): Seven studies (41.2%) examined primary psychotic disorders with comorbid substance use. While these studies provide valuable information about managing complex presentations, their applicability to substance-induced psychotic disorders specifically remains uncertain. All evidence for LAI antipsychotics and the majority of evidence for third-generation antipsychotics derives from this category.

This diagnostic stratification highlights the fundamental challenge in interpreting the literature on “substance-induced psychosis”: fewer than half of available studies examine the specific entity defined by DSM/ICD criteria, with the remainder examining either ambiguous presentations or explicitly different diagnostic entities. This heterogeneity has profound implications for clinical practice guidelines and future research direction

## 4. Discussion

### 4.1. The Evidence Landscape: Patterns of Knowledge and Neglect

This scoping review identified only 17 studies across four decades examining antipsychotic treatment in substance-induced psychotic disorders. The distribution of this limited evidence reveals stark imbalances: cannabis-induced and methamphetamine-induced psychoses account for over three-quarters of available studies (41.2% and 35.3%, respectively), while cocaine-induced and hallucinogen-induced presentations remain virtually uninvestigated despite their clinical significance.

Only three randomized controlled trials exist, both concentrated in cannabis and methamphetamine psychoses. Berk et al. (2000) [34] demonstrated equivalent efficacy between risperidone and haloperidol for cannabis-induced psychotic disorder in 30 patients meeting DSM-IV criteria, with risperidone showing fewer extrapyramidal side effects. Verachai et al. (2014) [35] compared quetiapine to haloperidol in 80 patients with methamphetamine-induced psychosis, demonstrating high remission rates for both medications (89% vs. 84%). Both trials provide Class I evidence that conventional antipsychotics—both first-generation and second-generation agents—effectively reduce acute psychotic symptoms in substance-induced presentations, though fundamental questions about optimal treatment duration, predictors of response, and long-term outcomes remain unaddressed.

Chuenchom et al.’s (2024) [38] retrospective cohort of 317 cannabis-induced psychosis patients meeting ICD-10 criteria provides the largest naturalistic outcome data available, showing that only 7% converted to schizophrenia at 1.3-year follow-up with appropriate treatment and abstinence. This finding challenges more pessimistic assumptions about substance-induced psychoses inevitably heralding chronic psychotic illness, though reconciling this relatively favorable prognosis with meta-analytic estimates of approximately 25% conversion rates raises important questions about diagnostic practices, patient populations, and treatment contexts across studies.

Evidence for third-generation antipsychotics in confirmed substance-induced psychotic disorders consists entirely of case reports: Ricci et al. [50] described complete recovery in four cannabis-induced psychosis patients treated with lurasidone, while Ricci et al. (2022) [39] reported improvement with cariprazine in one persistent methamphetamine-induced psychosis case. These preliminary observations suggest potential benefits but are insufficient for clinical recommendations. No studies have examined LAI formulations in substance-induced psychotic disorders meeting DSM/ICD criteria; all available LAI evidence [48,49] derives from dual disorder populations—primary psychotic disorders with comorbid substance use rather than substance-induced presentations.

The complete absence of controlled studies for cocaine-induced psychosis represents a particularly troubling gap given cocaine’s widespread use and well-characterized association with paranoid psychotic states. Clinicians managing cocaine psychosis must currently extrapolate from methamphetamine literature despite fundamental pharmacological differences. Similarly, no systematic treatment studies exist for hallucinogen-induced psychoses, an increasingly problematic gap as both classic psychedelics and novel psychoactive substances with hallucinogenic properties become more prevalent in clinical practice (Table 6).

### 4.2. Diagnostic Heterogeneity and Its Consequences for Evidence Interpretation

The diagnostic heterogeneity identified in this review represents more than a methodological limitation; it reflects fundamental conceptual challenges in how substance-induced psychotic disorders are studied and understood. Only 7 studies (41.2%) examined patients meeting strict DSM/ICD criteria for substance-induced psychotic disorder, while an equal proportion (41.2%) investigated dual disorders or first-episode psychosis with comorbid substance use—diagnostically distinct entities with different pathophysiology and prognosis. This distribution reveals a troubling pattern: most research ostensibly addressing “substance-induced psychosis” actually examines mixed populations where the distinction between substance-induced and primary psychotic presentations remains unclear or unstated.

The pervasive diagnostic ambiguity stems from inherent challenges in operationalizing DSM/ICD criteria in real-world research and clinical contexts. Temporal criteria requiring psychotic symptoms to develop during or shortly after substance use depend on retrospective patient recall in populations characterized by frequent polysubstance use, cognitive impairment during acute episodes, and limited insight. The persistence of symptoms beyond expected intoxication durations—typically specified as within one month—creates interpretive dilemmas that resist easy resolution: does prolonged psychosis reflect ongoing unreported use, individual vulnerability producing extended substance-induced states, or an emerging primary psychotic disorder unmasked or precipitated by substance exposure? These distinctions carry profound implications for treatment decisions, prognosis, and research interpretation, yet many studies provide insufficient detail about temporal relationships or diagnostic criteria application to resolve them.

The consequences of diagnostic ambiguity extend beyond individual clinical encounters to fundamentally shape research findings and their interpretation. Studies examining genetic polymorphisms associated with cannabis psychosis risk [43] face a persistent interpretive challenge: do identified markers predict vulnerability to substance-induced psychosis specifically, or do they simply reflect genetic associations with schizophrenia in populations that happen to use cannabis? Without clear diagnostic boundaries between substance-induced psychotic disorders and primary psychotic disorders with comorbid substance use, such genetic studies illuminate statistical associations but leave fundamental etiological questions unresolved. Similar challenges affect neuroimaging and biomarker investigations, where studies frequently examine mixed populations of cannabis users with psychotic disorders rather than diagnostically confirmed substance-induced presentations, fundamentally limiting conclusions about mechanisms specific to substance-induced psychotic disorders [53].

The conversion data from this review illustrates how diagnostic uncertainty affects prognostic understanding. Chuenchom et al.’s [38] finding that only 7% of patients with rigorously diagnosed cannabis-induced psychosis (ICD-10 criteria) converted to schizophrenia at 1.3-year follow-up stands in striking contrast to meta-analytic estimates of approximately 25% conversion reported in the broader literature. This discrepancy likely reflects multiple factors—diagnostic rigor in distinguishing substance-induced from emerging primary psychotic disorders, treatment intensity, sustained abstinence, underlying genetic vulnerability, cannabis potency and exposure patterns—yet their relative contributions remain inadequately characterized. Some patients experience single episodes resolving completely with abstinence and brief antipsychotic treatment, while others progress to chronic psychotic illness clinically indistinguishable from primary schizophrenia. Understanding what distinguishes these divergent trajectories represents a critical research priority with direct implications for both treatment decisions and prognostic counseling, yet progress requires the diagnostic clarity currently absent from much of the literature.

### 4.3. Treatment Evidence: Established Approaches and Unresolved Questions

The available evidence, despite significant limitations, supports several conclusions about antipsychotic treatment while highlighting critical knowledge gaps. The two randomized controlled trials—Berk et al. (2000) [34] for cannabis-induced psychosis and Verachai et al. (2014) [35] for methamphetamine-induced psychosis—demonstrated equivalent efficacy between typical and atypical antipsychotics. Both risperidone versus haloperidol (Berk et al.) and quetiapine versus haloperidol (Verachai et al.) [35] showed comparable symptom reduction, with second-generation agents offering fewer extrapyramidal side effects. This equivalence suggests that conventional dopamine blockade represents an effective therapeutic strategy for acute presentations, though whether this extends to longer-term outcomes, relapse prevention, or effects on substance use behaviors remains unexamined.

Critical treatment questions remain unanswered due to absence of systematic data. Treatment duration represents a particularly vexing issue: clinical experience suggests many substance-induced psychoses resolve within weeks to months with sustained abstinence, potentially not requiring extended antipsychotic treatment, yet no studies have systematically examined optimal treatment duration strategies. Patients presenting with first episodes, no family history of psychosis, and clear temporal relationships between substance use and symptom onset might reasonably receive time-limited treatment, while those with multiple episodes or symptoms persisting despite abstinence might warrant longer treatment pending diagnostic clarification. These decision-making approaches, however, rest more on clinical intuition than empirical evidence.

The evidence for third-generation antipsychotics in substance-induced psychotic disorders consists entirely of case reports: The first study [50] described four patients with cannabis-induced psychosis responding to lurasidone, while the second study [39] reported one methamphetamine-induced psychosis case improving with cariprazine. These medications’ unique receptor profiles—cariprazine’s preferential D3 receptor binding potentially affecting reward processing, lurasidone’s 5-HT7 antagonism potentially relevant for cognition and mood—offer theoretical advantages for addressing both psychotic symptoms and substance-related phenomena such as craving. However, the current evidence suffers from obvious limitations: absence of control groups, potential selective reporting of positive outcomes, extremely small sample sizes (n = 5 total across all published reports), and inability to establish causation or estimate effect sizes. Until adequately powered randomized controlled trials become available, claims about third-generation antipsychotic efficacy in substance-induced presentations remain speculative.

Long-acting injectable antipsychotics represent an area where theoretical rationale appears compelling yet empirical evidence specific to substance-induced psychoses is completely absent. The adherence challenges in populations characterized by ongoing substance use and chaotic lifestyles suggest potential benefits for LAI formulations. However, all available studies [48,49] examined LAI antipsychotics in first-episode psychosis with comorbid substance use or dual disorders—diagnostically distinct populations from substance-induced psychotic disorders. Several considerations further complicate LAI use: many substance-induced psychoses may be time-limited phenomena not requiring prolonged treatment, diagnostic uncertainty in early presentations complicates decisions about treatment intensity and duration, and concerns about therapeutic alliance in populations potentially ambivalent about psychiatric intervention. The applicability of findings from dual disorder studies to substance-induced presentations cannot be assumed given fundamental differences in pathophysiology, natural history, and prognosis.

### 4.4. Evidence Quality and the Limitations of Preliminary Data

The evidence base for antipsychotic treatment in substance-induced psychotic disorders is characterized by significant methodological limitations that constrain clinical inference. Only two randomized controlled trials exist [34,40], providing evidence exclusively for conventional antipsychotics (risperidone, haloperidol, quetiapine) in cannabis-induced and methamphetamine-induced psychosis.

For third-generation antipsychotics, the entire evidence base in substance-induced psychotic disorders consists of five patients across three case reports/series [39,50]. While these preliminary observations suggest potential efficacy, case reports without control groups cannot establish causation, rule out spontaneous remission, or provide meaningful estimates of treatment effect size. The methodological limitations of case reports are well-established: absence of comparison groups, lack of randomization, potential for publication bias favoring positive outcomes, and inability to control for confounding variables including concurrent interventions and natural disease course.

The situation for LAI antipsychotics is even more challenging: no studies whatsoever have examined these formulations in diagnostically confirmed substance-induced psychotic disorders. All available LAI evidence derives from primary psychotic disorders with comorbid substance use a fundamentally different diagnostic entity with distinct pathophysiology, prognosis, and treatment considerations.

These evidence gaps have important clinical implications. While case reports serve valuable hypothesis-generating functions and can alert clinicians to potential therapeutic options in treatment-resistant presentations, they cannot support clinical practice guidelines or standard-of-care recommendations. Extrapolating findings from dual disorder populations to substance-induced psychotic disorders is scientifically unjustified given the diagnostic and pathophysiological differences between these entities.

The field urgently requires adequately powered, randomized controlled trials of third-generation antipsychotics and LAI formulations specifically in diagnostically confirmed substance-induced psychotic disorders. Until such evidence becomes available, treatment decisions involving these agents must be approached cautiously, with careful individualized risk-benefit assessment and explicit acknowledgment of the limited evidence base.

### 4.5. Treatment Evidence and Unresolved Questions

The available evidence, despite significant limitations, supports several conclusions about antipsychotic treatment while simultaneously highlighting critical knowledge gaps that constrain clinical guidance. The two randomized controlled trials—Berk et al. (2000) [34] comparing risperidone to haloperidol in 30 patients with cannabis-induced psychotic disorder (DSM-IV criteria), and Verachai et al. (2014) [35] comparing quetiapine to haloperidol in 80 patients with methamphetamine-induced psychosis—demonstrated equivalent efficacy between typical and atypical antipsychotics. Both trials showed comparable rates of symptom reduction and remission (89% for quetiapine, 84% for haloperidol) with second-generation agents offering advantages in extrapyramidal side effect profiles but not superior efficacy. This equivalence suggests that conventional dopamine blockade represents an effective therapeutic strategy for acute substance-induced presentations, though fundamental questions remain unanswered: optimal treatment duration, predictors of response, relapse prevention strategies, and effects on continued substance use behaviors have not been systematically examined in any controlled studies.

Chuenchom et al.’s (2024) [38] cohort of 317 patients provides the largest naturalistic outcome data, demonstrating that appropriate antipsychotic treatment combined with abstinence can achieve favorable outcomes, with only 7% conversion to schizophrenia at 1.3-year follow-up. However, critical treatment questions remain unresolved. Treatment duration represents a particularly vexing issue where clinical practice rests more on intuition than empirical evidence. Many substance-induced psychoses appear to resolve within weeks to months with sustained abstinence, potentially not requiring extended antipsychotic treatment, yet no studies have systematically compared outcomes with different treatment duration strategies. Patients presenting with first episodes, no family history of psychosis, and clear temporal relationships between substance use and symptom onset might reasonably receive time-limited treatment with gradual discontinuation after symptom resolution and verified abstinence. Conversely, patients with multiple episodes, family history of psychotic disorders, or symptoms persisting despite documented abstinence might warrant longer treatment pending diagnostic clarification. These decision-making heuristics, however, rest on clinical reasoning rather than controlled comparisons, leaving clinicians without evidence-based guidance for one of the most consequential treatment decisions.

Evidence for third-generation antipsychotics in substance-induced psychotic disorders consists entirely of case reports insufficient for clinical recommendations. Ricci et al. (2022) [50] described complete recovery in four cannabis-induced psychosis patients treated with lurasidone 74–128 mg/day, while the same author [39] reported marked improvement with cariprazine in one persistent methamphetamine-induced psychosis case, including reduction in substance craving. These medications’ unique receptor profiles—cariprazine’s preferential D3 receptor binding potentially affecting reward processing and drug-seeking behavior, lurasidone’s potent 5-HT7 antagonism potentially relevant for cognition and mood—offer theoretical advantages for addressing both psychotic symptoms and substance-related phenomena. The preliminary observations suggest potential dual efficacy that would represent significant advances over conventional antipsychotics. However, the current evidence suffers from obvious limitations: total sample size of only five patients across all published reports, absence of control groups precluding causal inference, potential publication bias favoring positive outcomes, and inability to estimate treatment effect sizes or identify predictors of response. Until adequately powered randomized controlled trials become available, claims about third-generation antipsychotic efficacy in substance-induced presentations remain speculative rather than evidence-based.

Long-acting injectable antipsychotics represent an area where theoretical rationale appears compelling yet empirical evidence specific to substance-induced psychoses is completely absent. The rationale for LAI use seems persuasive: medication adherence represents a substantial challenge in populations characterized by ongoing substance use, cognitive impairment during acute episodes, chaotic lifestyles, and ambivalence about psychiatric treatment. Long-acting formulations ensure consistent medication delivery while providing structured clinical contact opportunities for monitoring and intervention. However, all available studies [48] examining 237 first-episode psychosis patients with substance use disorders; Szerman et al., 2020 [49] studying 40 patients with “dual disorders”) investigated populations with primary psychotic disorders and comorbid substance use rather than substance-induced psychotic disorders meeting DSM/ICD criteria. These represent diagnostically distinct entities with different pathophysiology, natural history, and prognosis. The applicability of findings from dual disorder studies—showing superior relapse-free survival with LAI formulations—cannot be assumed for substance-induced presentations. Moreover, several considerations complicate LAI use in substance-induced psychoses: many may be time-limited phenomena not requiring prolonged treatment, diagnostic uncertainty in early presentations complicates decisions about treatment intensity and duration, and concerns about therapeutic alliance in populations potentially ambivalent about psychiatric intervention require careful consideration.

## 5. Conclusions

This scoping review documents a field requiring not merely incremental additions to existing knowledge but rather fundamental expansion into unstudied territories and methodological innovation to address inherent complexities. The increasing prevalence of substance-induced psychoses, the emergence of novel psychoactive substances with unpredictable psychotomimetic potential, and the substantial personal and societal burden associated with these conditions make continued research investment essential. By comprehensively mapping the current evidence landscape and explicitly identifying research priorities, this scoping review provides foundation for advancing toward more complete, evidence-based approaches to managing substance-induced psychotic disorders across their full spectrum. The path forward requires sustained commitment to addressing the challenging methodological and practical barriers that have contributed to current evidence gaps, with research prioritization reflecting clinical burden and public health significance rather than convenience or historical precedent.

## Figures and Tables

**Table 1:** Quality assessment summary.

Study	Design	Diagnostic Category	Quality Assessment Tool	Overall Quality	Key Strengths	Key Limitations
RANDOMIZED CONTROLLED TRIALS						
Berk et al., 2000 [34]	RCT	DSM/ICD Criteria	RoB 2	Low risk	Double-blind; ITT analysis; low attrition (10%); DSM-IV criteria	Small sample (n = 30); single-center
Verachai et al., 2014 [35]	RCT	DSM/ICD Criteria	RoB 2	Low risk	Computer randomization; double-blind; low attrition (7.5%)	Small sample (n = 80); 4-week follow-up
OBSERVATIONAL STUDIES—DSM/ICD CRITERIA						
Chuenchom et al., 2024 [38]	Cohort	DSM/ICD Criteria	NOS	8/9 stars	Large sample (n = 317); ICD-10 criteria; 1.3-year follow-up	Retrospective; single-center; incomplete attrition data
OBSERVATIONAL STUDIES—MIXED PRESENTATIONS						
Gouse et al., 2023 [40]	Cross-sectional	Mixed Presentations	NOS	5/9 stars	Large sample (n = 2134); ED-based	No diagnostic verification; unclear case definition; no follow-up
Rafizadeh et al., 2023 [41]	Observational	Mixed Presentations	NOS	6/9 stars	Adequate sample (n = 87)	Unclear diagnostic boundaries; brief follow-up
Paulus et al., 2005 [42]	Cohort	Mixed Presentations	NOS	6/9 stars	Neuroimaging biomarkers	Very small sample (n = 17); diagnostic ambiguity
OBSERVATIONAL STUDIES—DUAL DISORDERS						
Di Forti et al., 2012 [43]	Case–control	Dual Disorders	NOS	7/9 stars	Large sample (n = 767); genetic analysis	Mixed population; not pure SIPD
van Winkel et al., 2011 [44]	Observational	Dual Disorders	NOS	7/9 stars	Very large sample (n = 1120)	Primary psychosis + cannabis; not SIPD
Blasi et al., 2011 [45]	Observational	Dual Disorders	NOS	6/9 stars	Genetic + neuroimaging	Mixed diagnostic population
Chiappini et al., 2024 [46]	Observational	Dual Disorders	NOS	5/9 stars	Dual diagnosis focus	Explicitly dual disorders, not SIPD; small sample (n = 24)
Cavallotto et al., 2024 [47]	Observational	Dual Disorders	NOS	5/9 stars	SUD population	Not SIPD; small sample (n = 23)
Abdel-Baki et al., 2019 [48]	Cohort	Dual Disorders	NOS	7/9 stars	Large sample (n = 237); 3-year follow-up	FEP + SUD, not SIPD
Szerman et al., 2020 [49]	Observational	Dual Disorders	NOS	6/9 stars	Dual disorders focus	Not SIPD; small sample (n = 40)
CASE SERIES/REPORTS						
Ricci et al., 2022 [50]	Case series	DSM/ICD Criteria	Clinical documentation	Adequate	Clear diagnostic criteria; complete recovery documented	No controls; n = 4; publication bias risk
Roberto et al., 2016 [51]	Case series	DSM/ICD Criteria	Clinical documentation	Adequate	Synthetic cannabinoids focus	No controls; brief follow-up
Seddigh et al., 2014 [52]	Case series	DSM/ICD Criteria	Clinical documentation	Adequate	Treatment-resistant cases; clozapine response	No controls; n = 2; 8–9 month follow-up
Ricci et al., 2022 [39]	Case report	DSM/ICD Criteria	Clinical documentation	Adequate	Clear documentation; persistent psychosis	Single case; no generalizability

**Table 2:** Characteristics of included studies (n = 17).

Study	Year	Country	Design	n	Substance	Population
SUBSTANCE-INDUCED PSYCHOTIC DISORDERS (DSM/ICD CRITERIA)						
Berk et al. [34]	2000	Australia	RCT	30	Cannabis	Cannabis-induced psychotic disorder (DSM-IV)
Chuenchom et al. [38]	2024	Thailand	Cohort	317	Cannabis	Cannabis-induced psychosis (ICD-10)
Ricci et al. [50]	2022	Italy	Case series	4	Cannabis	Cannabis-induced psychosis
Roberto et al. [51]	2016	Italy	Case series	Multiple	Synthetic cannabinoids	FEP after synthetic cannabinoid use
Verachai et al. [35]	2014	Thailand	RCT	80	Methamphetamine	METH-induced psychosis
Seddigh et al. [52]	2014	Iran	Case series	2	Methamphetamine	Treatment-resistant METH psychosis
Ricci et al. [39]	2022	Italy	Case report	1	Methamphetamine	Persistent METH-induced psychosis
MIXED PRESENTATIONS (SUBSTANCE USE + PSYCHOTIC SYMPTOMS, AMBIGUOUS BOUNDARIES)						
Gouse et al. [40]	2023	South Africa	Cross-sectional	2134	Cannabis	Acute psychosis with THC+ screen
Rafizadeh et al. [41]	2023	Iran	Observational	87	Methamphetamine	Methamphetamine use disorder
Paulus et al. [42]	2005	USA	Cohort	17	Methamphetamine	METH users with psychotic symptoms
DUAL DISORDERS/PRIMARY PSYCHOSIS + SUBSTANCE USE						
Di Forti et al. [43]	2012	UK	Case–control	767	Cannabis	Cannabis users ± psychotic disorder
van Winkel et al. [44]	2011	The Netherlands	Observational	1120	Cannabis	Psychotic patients + cannabis use
Blasi et al. [45]	2011	Italy	Observational	256	Mixed	Psychotic disorder patients
Chiappini et al. [46]	2024	Italy	Observational	24	Mixed	“Dual disorders”
Cavallotto et al. [47]	2024	Italy	Observational	23	Mixed	“Substance use disorders”
Abdel-Baki et al. [48]	2019	Canada	Cohort	237	Mixed	First-episode psychosis + SUD
Szerman et al. [49]	2020	Spain	Observational	40	Mixed	“Dual disorders”

**Table 3:** Evidence distribution by substance type and diagnostic category.

Substance Category	DSM/ICD Criteria	Mixed Presentations	Dual Disorders	Total Studies	Percentage	Evidence Quality
Cannabis-induced psychosis	4	1	2	7	41.2%	Moderate
Methamphetamine-induced	3	2	0	5	29.4%	Moderate
Cocaine-induced psychosis	0	0	0	0	0%	NO EVIDENCE
Other stimulants	0	0	0	0	0%	NO EVIDENCE
Hallucinogens	0	0	0	0	0%	NO EVIDENCE
Mixed substances (dual disorders/FEP + SUD)	0	0	5	5	29.4%	Limited
TOTAL	7	3	7	17	100%	-

Note: Only 3 RCTs identified (17.6% of studies), all examining substance-induced psychotic disorders meeting DSM/ICD criteria. Complete absence of studies for cocaine, other stimulants, and hallucinogens. Evidence for rigorously diagnosed substance-induced psychotic disorders concentrated in cannabis (57% of DSM/ICD criteria studies) and methamphetamine (43% of DSM/ICD criteria studies).

**Table 4:** Treatment outcomes—cannabis-related psychosis stratified by diagnostic category substance-induced psychotic disorder (DSM/ICD criteria).

Medication	Study	Design	n	Dose	Outcome	Key Findings
Risperidone	Berk, 2000 [34]	RCT	15	4–6 mg/day (mean 4.8 mg/day)	Equivalent to haloperidol	Fewer EPS; Class I evidence; BPRS, CGI, GAF improvements
Haloperidol	Berk, 2000 [34]	RCT	15	5–20 mg/day (mean 11.3 mg/day)	Equivalent to risperidone	More EPS; Class I evidence; equal efficacy on psychotic symptoms
Risperidone	Chuenchom, 2024 [38]	Cohort	265	2–6 mg/day (variable dosing)	Clinical improvement	7% converted to schizophrenia at 1.3 years; 93% favorable outcome with abstinence
Haloperidol	Chuenchom, 2024 [38]	Cohort	63	5–15 mg/day (variable dosing)	Clinical improvement	Used less frequently; similar effectiveness; ICD-10 diagnosed cases
Perphenazine	Chuenchom, 2024 [38]	Cohort	25	8–24 mg/day	Clinical improvement	Minority of patients; adequate response
Lurasidone	Ricci, 2022 [50]	Case series	4	74–128 mg/day (mean 96 mg/day)	Complete recovery (100%)	No significant adverse effects; preliminary evidence; third-generation AP
**MIXED PRESENTATIONS (AMBIGUOUS DIAGNOSTIC BOUNDARIES)**						
**Intervention**	**Study**	**Design**	**n**	**Details**	**Outcome**	**Key Findings**
Parenteral antipsychotics + benzodiazepines	Gouse, 2023 [40]	Cross-sectional	2134	Various agents (IM haloperidol, IM olanzapine, IM lorazepam most common)	Emergency stabilization	THC+ patients required more restraints and parenteral medications; no difference in hospitalization rates or 90-day ED returns
**PRIMARY PSYCHOSIS + CANNABIS USE (DUAL DISORDERS)**						
**Study Focus**	**Study**	**Design**	**n**	**Intervention/Assessment**	**Finding**	**Key Findings**
Genetic markers (AKT1)	Di Forti, 2012 [43]	Case-control	767	AKT1 rs2494732 genotyping	Risk prediction	CC genotype: OR > 2 for cannabis-associated psychosis; unclear if SIPD-specific or schizophrenia association
Cognitive effects moderated by genetics	van Winkel, 2011 [44]	Observational	1120	AKT1 genotype + cognitive testing	AKT1 moderation	Cannabis effects on cognition vary by genotype; psychotic patients + cannabis use, not pure SIPD
DRD2/AKT1 interaction	Blasi, 2011 [45]	Observational	256	Genetic analysis + olanzapine response	Pharmacogenetic response	DRD2/AKT1 interactions predict olanzapine response; psychotic disorder population

Evidence quality: Case series/reports provide preliminary observations only and cannot support clinical recommendations; findings should be interpreted with extreme caution.

**Table 5:** Treatment outcomes—methamphetamine-related psychosis stratified by diagnostic category.

Medication	Study	Design	n	Dose	Outcome	Key Findings
Quetiapine	Verachai, 2014 [35]	RCT	40	~400 mg/day (mean 375 mg/day)	89% remission	Equivalent to haloperidol; Class I evidence; well tolerated
Haloperidol	Verachai, 2014 [35]	RCT	40	10–15 mg/day (mean 12.5 mg/day)	84% remission	Equivalent to quetiapine; Class I evidence; more EPS
Clozapine	Seddigh, 2014 [52]	Case series	2	200–300 mg/day	Complete resolution	Response within 2 weeks; treatment-resistant cases; sustained benefit 8–9 months
Cariprazine	Ricci, 2022 [39]	Case report	1	3–6 mg/day (titrated)	Marked improvement	Reduced craving; improved positive and negative symptoms; treatment-resistant case
**MIXED PRESENTATIONS (AMBIGUOUS DIAGNOSTIC BOUNDARIES)**						
**Medication/Intervention**	**Study**	**Design**	**n**	**Details**	**Outcome**	**Key Findings**
Clozapine	Rafizadeh, 2023 [41]	Observational	87	200–450 mg/day (variable dosing)	Increased abstinence	OR = 3.05 for abstinence; RR = 0.45 for relapse; methamphetamine use disorder population
fMRI biomarkers	Paulus, 2005 [42]	Cohort	17	N/A (predictive study)	Relapse prediction	94% sensitivity, 86% specificity for predicting psychosis relapse; diagnostic ambiguity present

Evidence quality: Case series/reports provide preliminary observations only and cannot support clinical recommendations; findings should be interpreted with extreme caution.

**Table 6:** Critical evidence gaps.

Domain	Available Evidence	Critical Gap	Priority Level
Cannabis psychosis	1 RCT, 1 large cohort, case series	Synthetic cannabinoids; long-term outcomes; third-generation RCTs	HIGH
Methamphetamine psychosis	1 RCT, limited observational	Treatment-resistant protocols; long-term follow-up	HIGH
Cocaine psychosis	NONE	All aspects—no controlled studies	CRITICAL
Other stimulants (MDMA, cathinones)	NONE	All aspects—no controlled studies	CRITICAL
Hallucinogens (LSD, ketamine, PCP)	NONE	All aspects—no controlled studies	CRITICAL
Novel psychoactive substances	1 case series only	Systematic investigation needed	CRITICAL
Third-generation antipsychotics	Case reports only	RCTs with clear diagnostic criteria	HIGH
LAI formulations	Indirect evidence	Studies in pure substance-induced psychosis	MODERATE
Treatment resistance	2 case reports	Systematic protocols and definitions	HIGH

RCT = Randomized Controlled Trial; LAI = Long-Acting Injectable.

## Data Availability

No new data were created or analyzed in this study.

## Supplementary Materials

The following supporting information can be downloaded at: https://www.mdpi.com/article/10.3390/healthcare13243210/s1.
