# Supplementary material for: Substance-Specific Treatment Responses and Resistance Patterns in Induced Psychoses: A Scoping Review of Antipsychotic Efficacy
Source: Healthcare (Basel). 2025 Dec 8;13(24):3210. doi: 10.3390/healthcare13243210 (PMC12733067; doi:10.3390/healthcare13243210)
Supplement: Supplementary file 1 [file healthcare-13-03210-s001.zip › healthcare-4012930-supplementary.pdf]

Appendix A

Appendix A: Protocol Deviations and Sensitivity Analyses

Comparison Between Registered Protocol and Final Methodology

Protocol Element	Original PROSPERO Registration	Final Implementation	Deviation Rationale	Impact Assessment
**Primary Analysis**	Comprehensive meta-analysis using random-effects models	Qualitative synthesis with limited quantitative analysis	Study design heterogeneity precluded valid statistical pooling	Enhanced methodological rigor; maintained clinical relevance
**Heterogeneity Management**	Meta-regression to explore sources of heterogeneity	Stratified analysis by study design and substance type	Insufficient homogeneous studies for meta-regression	More transparent presentation of study differences
**Effect Measures**	Standardized mean differences (SMD) and odds ratios	Response rates with confidence intervals, descriptive statistics	Outcome heterogeneity prevented standardized effect calculations	Better reflection of clinical reality
**Subgroup Analyses**	Pre-planned by substance type, antipsychotic class, age groups	Substance type and study design stratification only	Limited data availability for all planned subgroups	Focused on most clinically relevant comparisons
**Publication Type**	Peer-reviewed journal articles only	Included conference abstracts with sensitivity analysis	Expanded scope to capture emerging evidence	Addressed through separate quality assessment
**Language Restriction**	English language only	English language only	No deviation	Acknowledged as limitation
**Risk of Bias Tools**	Cochrane RoB 2 for RCTs, Newcastle-Ottawa for observational studies	Added AACODS for grey literature, modified checklist for case series	Expanded scope required additional assessment tools	Comprehensive quality evaluation
**Statistical Software**	RevMan 5.4	R Studio with meta and metafor packages	Enhanced analytical capabilities needed	More sophisticated statistical analysis

Sensitivity Analyses Performed

Analysis Type	Studies Included	Studies Excluded	Primary Finding	Impact on Conclusions
**Study Design**				
**RCTs only (n=5)**	Berk et al., Schnell et al., Verachai et al., Robinson et al., Hamidovic et al.	All observational studies and case series	Response rate: 76% (95% CI: 71-81%)	Higher response rates than mixed analysis
**Prospective studies only (n=10)**	All cohort and longitudinal studies	RCTs, case series, cross-sectional	Response rate: 67% (95% CI: 65-69%)	More conservative estimates
**Publication Type**				
**Peer-reviewed only (n=22)**	All journal publications	Conference abstracts (n=3)	No substantial difference in conclusions	Conference abstracts did not bias results
**Geographic Distribution**				
**North America/Europe (n=20)**	Studies from developed healthcare systems	Asian/Other regions (n=5)	Slightly higher response rates (71% vs 66%)	Limited impact on generalizability
**Sample Size**				

**Large studies (>100 participants)**	8 studies	Small studies (<100 participants)	More conservative response rates (68% vs 74%)	Small study bias confirmed

Quality Assessment Impact

Quality Metric	High Quality Studies	Moderate Quality	Low Quality	Effect on Synthesis
**RCT Quality (RoB 2)**	3 studies (Low risk)	2 studies (Some concerns)	0 studies (High risk)	Strong evidence base for controlled comparisons
**Observational Quality (NOS)**	6 studies (≥7 points)	8 studies (5-6 points)	1 study (<5 points)	Generally high quality observational evidence
**Grey Literature (AACODS)**	2 abstracts (≥8 points)	1 abstract (6-7 points)	0 abstracts (<6 points)	Acceptable quality for inclusion
**Case Series Quality**	3 studies (Adequate reporting)	2 studies (Limited reporting)	0 studies (Poor reporting)	Descriptive value maintained

Protocol Adherence Assessment

PROSPERO Registration Requirement	Adherence Status	Justification
**Pre-specified inclusion/exclusion criteria**	✓ Fully adhered	Criteria applied as registered
**Comprehensive search strategy**	✓ Fully adhered	All databases searched as planned
**Duplicate screening by two reviewers**	✓ Fully adhered	Independent screening completed
**Risk of bias assessment**	✓ Enhanced	Additional tools added for study types encountered
**Data extraction protocols**	✓ Fully adhered	Standardized forms used
**Statistical analysis plan**	⚠ Modified	Changed due to data heterogeneity
**Subgroup analyses**	⚠ Partially completed	Limited by available data
**Sensitivity analyses**	✓ Enhanced	Additional analyses beyond protocol

Legend: ✓ = Full adherence or enhancement ⚠ = Justified modification ✗ = Deviation requiring explanation **Transparency Statement:** All deviations from the registered protocol were made prior to data analysis and were driven by methodological considerations to ensure scientific rigor. The transition from meta-analysis to qualitative synthesis represents a methodological improvement rather than a compromise, as it avoids the inappropriate pooling of heterogeneous studies that would have produced misleading results.

APPENDIX B PRISMA-ScR Checklist

SECTION	ITEM	CHECKLIST ITEM	REPORTED ON PAGE
TITLE	1	Identify the report as a scoping review.	Page 1
**ABSTRACT**			
Structured summary	2	Provide a structured summary including background, objectives, eligibility criteria, sources of evidence, charting methods, results, and conclusions.	Page 1
**INTRODUCTION**			
Rationale	3	Describe the rationale for the review in context of what is known. Explain why the review questions lend themselves to a scoping review.	Pages 2-4, Section 1
Objectives	4	Provide explicit statement of questions and objectives with reference to key elements (population, concepts, context).	Page 4, Sections 1 and 2.2
**METHODS**			

Protocol	5	Indicate if protocol exists, where it can be accessed, and registration information.	Page 4, Section 2.1: PROSPERO CRD420251123724
Eligibility criteria	6	Specify characteristics used as eligibility criteria with rationale.	Pages 5-6, Section 2.4
Information sources	7	Describe all information sources and search dates.	Page 5, Section 2.3: 5 databases, Jan 1985-Aug 2025
Search	8	Present full electronic search strategy for at least one database.	Page 5, Section 2.3
Selection	9	State the process for selecting sources of evidence.	Page 6, Section 2.5: Dual screening VR/GM, SC adjudication
Data charting	10	Describe methods of charting data from included sources.	Page 6, Section 2.6: Standardized form, pilot-tested
Data items	11	List and define all variables for which data were sought.	Page 6, Section 2.6
Critical appraisal	12	If done, provide rationale and describe methods used.	Pages 6-7, Section 2.7: RoB 2, NOS, modified checklist
Synthesis	13	Describe methods of handling and summarizing charted data.	Page 7, Section 2.8: Descriptive analysis
**RESULTS**			
Selection	14	Give numbers screened, assessed, included, with reasons for exclusions, using flow diagram.	Page 7, Section 3.1 and Figure 1
Characteristics	15	For each source, present characteristics and citations.	Pages 7-9, Tables 2-3, Section 3.1
Critical appraisal	16	If done, present data on critical appraisal.	Page 7, Table 1
Results individual	17	For each source, present relevant charted data.	Pages 9-14, Sections 3.3-3.8, Tables 4-5
Synthesis	18	Summarize charting results as they relate to review questions.	Pages 9-14, Narrative synthesis by substance
**DISCUSSION**			
Summary	19	Summarize main results, link to objectives, consider relevance.	Pages 14-23, Sections 4.1-4.5
Limitations	20	Discuss limitations of the scoping review process.	Pages 22-23, Section 4.5
Conclusions	21	Provide general interpretation with potential implications.	Pages 23-24, Section 5
**FUNDING**			
Funding	22	Describe sources of funding for review.	Page 25: No specific grant funding

Appendix C Complete Search Strings by Database

Database	Search Date	Interface	Results	Complete Search String
**PubMed/MEDLINE**	June 15, 2025	PubMed (https://pubmed.ncbi.nlm.nih.gov)	1,247	("substance-induced psychosis"[Title/Abstract] OR "substance-induced psychotic disorder"[Title/Abstract] OR "drug-induced psychosis"[Title/Abstract] OR "cannabis psychosis"[Title/Abstract] OR "cannabis-induced psychosis"[Title/Abstract] OR "methamphetamine psychosis"[Title/Abstract] OR "stimulant psychosis"[Title/Abstract] OR "cocaine psychosis"[Title/Abstract] OR "hallucinogen psychosis"[Title/Abstract] OR "amphetamine psychosis"[Title/Abstract]) AND ("antipsychotic"[Title/Abstract] OR "neuroleptic"[Title/Abstract] OR "antipsychotic agents"[MeSH] OR "risperidone"[Title/Abstract] OR "haloperidol"[Title/Abstract] OR "quetiapine"[Title/Abstract] OR "olanzapine"[Title/Abstract] OR "aripiprazole"[Title/Abstract] OR "clozapine"[Title/Abstract] OR "lurasidone"[Title/Abstract] OR "cariprazine"[Title/Abstract] OR "brexpiprazole"[Title/Abstract] OR "paliperidone"[Title/Abstract]) AND ("treatment"[Title/Abstract] OR "therapy"[Title/Abstract] OR "management"[Title/Abstract] OR "response"[Title/Abstract] OR "efficacy"[Title/Abstract] OR "outcome"[Title/Abstract] OR "treatment outcome"[MeSH])
**Embase**	June 18, 2025	Ovid (Embase 1947-June 2025)	892	('substance induced psychosis'/exp OR 'drug induced psychosis':ti,ab OR 'cannabis psychosis'/exp OR 'cannabis induced psychosis':ti,ab OR 'methamphetamine psychosis':ti,ab OR 'stimulant psychosis':ti,ab OR 'cocaine psychosis':ti,ab OR 'hallucinogen psychosis':ti,ab OR 'amphetamine psychosis':ti,ab) AND ('antipsychotic agent'/exp OR antipsychotic:ti,ab OR neuroleptic:ti,ab OR 'risperidone'/exp OR 'haloperidol'/exp OR 'quetiapine'/exp OR 'olanzapine'/exp OR 'aripiprazole'/exp OR 'clozapine'/exp OR 'lurasidone'/exp OR 'cariprazine'/exp OR 'brexpiprazole'/exp) AND ('treatment'/exp OR 'drug therapy'/exp OR therapy:ti,ab OR management:ti,ab OR 'treatment response'/exp OR efficacy:ti,ab OR 'treatment outcome'/exp)
**PsycINFO**	June 20, 2025	EBSCOhost (1806-June Week 2 2025)	456	(TI ("substance-induced psychosis" OR "drug-induced psychosis" OR "cannabis psychosis" OR "cannabis-induced psychosis" OR "methamphetamine psychosis" OR "stimulant psychosis" OR "cocaine psychosis" OR "hallucinogen psychosis") OR AB ("substance-induced psychosis" OR "drug-induced psychosis" OR "cannabis psychosis" OR "cannabis-induced psychosis" OR "methamphetamine psychosis" OR "stimulant psychosis" OR "cocaine psychosis" OR "hallucinogen psychosis")) AND (TI (antipsychotic OR neuroleptic OR risperidone OR haloperidol OR quetiapine OR olanzapine OR aripiprazole OR clozapine OR lurasidone OR cariprazine OR brexpiprazole) OR AB (antipsychotic OR neuroleptic OR risperidone OR haloperidol OR quetiapine OR olanzapine OR aripiprazole OR clozapine OR lurasidone OR cariprazine OR brexpiprazole)) AND (TI (treatment OR therapy OR management OR response OR efficacy OR outcome) OR AB (treatment OR therapy OR management

				OR response OR efficacy OR outcome))
**Web of Science**	June 22, 2025	Core Collection (Clarivate)	523	TS=("substance-induced psychosis" OR "drug-induced psychosis" OR "cannabis psychosis" OR "cannabis-induced psychosis" OR "methamphetamine psychosis" OR "stimulant psychosis" OR "cocaine psychosis" OR "hallucinogen psychosis") AND TS=(antipsychotic OR neuroleptic OR risperidone OR haloperidol OR quetiapine OR olanzapine OR aripiprazole OR clozapine OR lurasidone OR cariprazine OR brexpiprazole) AND TS=(treatment OR therapy OR management OR response OR efficacy OR outcome)
**Cochrane Library**	June 25, 2025	Wiley (Issue 6, 2025)	229	("substance-induced psychosis" OR "drug-induced psychosis" OR "cannabis psychosis" OR "methamphetamine psychosis"):ti,ab,kw AND (antipsychotic OR neuroleptic OR risperidone OR haloperidol OR quetiapine OR olanzapine OR aripiprazole OR clozapine OR lurasidone OR cariprazine):ti,ab,kw

Total unique citations after deduplication: 2,347

Table C2. Grey Literature and Supplementary Search Details

**Source**	**Search Date**	**Search Terms**	**Results**
ClinicalTrials.gov	June 28, 2025	"substance-induced psychosis" AND "antipsychotic"	12 trials
WHO ICTRP	June 28, 2025	"substance-induced psychosis" AND "antipsychotic"	8 trials
ProQuest Dissertations & Theses	June 30, 2025	"substance-induced psychosis" AND "antipsychotic treatment"	34 dissertations
APA Annual Meeting Abstracts	July 5, 2025	Manual search 2019-2024 proceedings	18 abstracts reviewed
ECNP Congress Abstracts	July 8, 2025	Manual search 2019-2024 proceedings	22 abstracts reviewed
SIRS Conference Abstracts	July 10, 2025	Manual search 2019-2024 proceedings	14 abstracts reviewed
Reference list searches	July 15-30, 2025	Manual review of all included studies	45 additional citations screened
Forward citation tracking (Google Scholar)	August 5-12, 2025	Berk 2000, Verachai 2014	127 citing articles screened

Note: TS=Topic Search; TI=Title; AB=Abstract; MeSH=Medical Subject Headings; exp=exploded search term
